# Fractional Stability of Trunk Acceleration Dynamics of Daily-Life Walking: Toward a Unified Concept of Gait Stability

**DOI:** 10.3389/fphys.2017.00516

**Published:** 2017-08-29

**Authors:** Espen A. F. Ihlen, Kimberley S. van Schooten, Sjoerd M. Bruijn, Mirjam Pijnappels, Jaap H. van Dieën

**Affiliations:** ^1^Department of Neuromedicine and Movement Science, Norwegian University of Science and Technology (NTNU) Trondheim, Norway; ^2^Department of Biomedical Kinesiology and Physiology, Simon Fraser University Burnab, BC, Canada; ^3^Centre for Hip Health and Mobility, University of British Columbia Vancouver, BC, Canada; ^4^Department of Human Movement Sciences, MOVE Research Institute Amsterdam, Vrije Universiteit Amsterdam Amsterdam, Netherlands

**Keywords:** Lyapunov exponent, walking dynamics, accidental falls, aged 65 and over, fractional calculus

## Abstract

Over the last decades, various measures have been introduced to assess stability during walking. All of these measures assume that gait stability may be equated with exponential stability, where dynamic stability is quantified by a Floquet multiplier or Lyapunov exponent. These specific constructs of dynamic stability assume that the gait dynamics are time independent and without phase transitions. In this case the temporal change in distance, *d*(*t*), between neighboring trajectories in state space is assumed to be an exponential function of time. However, results from walking models and empirical studies show that the assumptions of exponential stability break down in the vicinity of phase transitions that are present in each step cycle. Here we apply a general non-exponential construct of gait stability, called fractional stability, which can define dynamic stability in the presence of phase transitions. Fractional stability employs the fractional indices, α and β, of differential operator which allow modeling of singularities in *d*(*t*) that cannot be captured by exponential stability. The fractional stability provided an improved fit of *d*(*t*) compared to exponential stability when applied to trunk accelerations during daily-life walking in community-dwelling older adults. Moreover, using multivariate empirical mode decomposition surrogates, we found that the singularities in *d*(*t*), which were well modeled by fractional stability, are created by phase-dependent modulation of gait. The new construct of fractional stability may represent a physiologically more valid concept of stability in vicinity of phase transitions and may thus pave the way for a more unified concept of gait stability.

## Introduction

The number of studies on gait stability has rapidly increased during the last two decades (as evidenced by two review papers Hamacher et al., 2011; Bruijn et al., 2013). Despite this increase in interest, a lack of consensus remains regarding the definition of gait stability and the numerical operationalization. Current definitions of gait stability refer to the resistance of the gait kinematics to disturbances or the ability to recover gait kinematics after perturbations, e.g., “*the ability to maintain functional locomotion despite the presence of small kinematic disturbances or control errors*” (p. 172, England and Granata, 2007) and “*gait that does not lead to falls in spite of perturbations*” (p. 2, Bruijn et al., 2013). Thus, the main aim of most studies is to develop or utilize numerical measures that allow detecting instability during walking among fall-prone older persons and patients with neurodegenerative diseases (e.g., Dingwell and Cusumano, 2000; Toebes et al., 2012).

There are two dominant theoretical approaches of stability called *dynamic stability* and *structural stability*. Dynamic stability is based on the theory of stability of dynamical systems created by Alexandr M. Lyapunov in 1892, and published in his thesis “*General problem of the stability of motion*” (Lyapunov, 1992). Lyapunov's constructs of dynamic stability assesses the sensitivity of a mechanical system to small perturbations and is often used to quantify how kinematics of our walking pattern change in response to small perturbations. A particular part of Lyapunovs' framework of dynamic stability, called exponential stability, has been used in gait analysis to assess the “*local dynamic stability*” of gait kinematics (Dingwell and Cusumano, 2000). According to the construct of exponential stability, the reaction size *d*(*t)* of the gait dynamics to an infinitesimal perturbation of size *d*(0) is an exponential function of time *t, d*(*t*) = *d*(0)exp(λ*t*). The reaction size *d*(*t*) and the corresponding Lyapunov exponent λ can be assessed for each degree of freedom of the gait dynamics (i.e., each direction of the state space), but usually the largest Lyapunov exponent is assessed. A positive Lyapunov exponent, λ > 0, indicates that initially small perturbations grow exponentially with *t* (i.e., an instable system), whereas a negative exponent, λ < 0, indicates that small perturbations decrease exponentially with *t* (i.e., a stable system). The concept of exponential stability has been used to assess gait stability in several patient groups, experimental perturbation studies, walking models, and daily-life walking (Dingwell et al., 2000; Buzzi et al., 2003; Su and Dingwell, 2007; Kurz et al., 2010; McAndrew et al., 2011; Roos and Dingwell, 2011; van Schooten et al., 2011; Bruijn et al., 2012; Hamacher et al., 2016; van Schooten et al., 2016; de Melker Worms et al., 2017).

A second theoretical approach of stability of dynamical systems, called structural stability, was introduced by Andronov and Pontrjagin (1937). It was further implemented as a main theory of human motor control by Kelso (1995), inspired by Haken's (1977) framework of *synergetics*, and Thom's (1975) theory of structural instabilities and morphogenesis. This theory considers gait dynamics to be structurally unstable when its intrinsic exponential stability characteristics change by a small perturbation of its topology (i.e., structure). The changes of the exponential stability characteristics are referred to as phase transitions and appear at λ = 0, when λ switches to either negative or positive (i.e., stable or unstable, respectively). Haken (1977) and later Kelso (1995) suggested that the degrees of freedom (DOFs) of the human neuromuscular system couple into coordinative structures, or synergies, to adapt the movements to heterogeneous and ever changing surroundings. Haken (1977, 1983) and Kelso (1995) conjectured that coordinated motion observed in nature is generated by structurally unstable systems. In structurally unstable systems, a small number of observable DOF with λ close to 0, called *order parameters*, will *enslave* all other DOFs with λ > 0 and λ < 0. According to Lyapunov's theory of dynamic stability above, DOFs with λ > 0 will diverge exponentially into highly complicated and irregular movement, whereas DOFs with λ < 0 will converge exponentially into a very simplistic periodic motion, neither of which represent human movement. Thus, according to theoretical approach of structural stability, transitions in exponential stability are important to allow the gait dynamics to adapt to changing walking contexts.

According to the bifurcation theory of structurally unstable systems, the reaction size *d*(*t*) to a small perturbation will be a non-exponential function of time (Kuznetsov, 2004). Thus, the construct of exponential stability will no longer be valid. The presence of a non-exponential reaction size *d*(*t*) was shown in several previous studies on gait stability including Figure 7 in the original work by Dingwell and Cusumano (2000) (see also van Schooten et al., 2013). Eventhough Kelso (1995) introduced a methodology to investigate structural changes in the dynamics of bimanual coordination tasks, no consistent methodology has been introduced to assess stability of coordinative patterns, such as human gait, displaying phase transitions. Such methodology would have to generalize the construct of exponential stability to be valid in both structurally stable and unstable dynamics and thereby obtain a single coherent concept of gait stability.

The main aim of the present article is to apply a new and general construct of stability to gait. This concept, called fractional stability, extends the construct of exponential stability to structurally unstable systems in the presence of phase transitions. We will illustrate how the construct of fractional stability can be applied to real-life walking data obtained with a trunk-worn accelerometer in community-dwelling older adults.

## Methods

In this extended methods section, we first shortly review the construct of exponential stability and discuss how exponential stability is usually assessed in gait analysis (Section Dynamic stability of human gait). Then we explain how exponential stability breaks down in the presence of phase transitions and how the general construct of fractional stability could still quantify dynamic stability (Section Fractional stability and phase transitions in human gait). Finally, we show a proof-of-concept on how the construct of fractional stability can be employed to trunk accelerometer data of daily-life walking in community-dwelling older adults (Sections Fractional stability in trunk acceleration dynamics of community dwelling older persons: A proof-of-concept study).

### Dynamic stability of human gait

Gait dynamics could be mathematically expressed by the solution of an unknown and complex equation of motion. In gait analysis, the gait dynamics is reconstructed from the measured movement kinematics *x*(*t*) by delayed coordinate embedding (Takens, 1980; Sauer et al., 1991):

(1) $$\begin{array}{r} \text{x}\left(t\right)=\left[x\left(t\right),x\left(t+l\right),x\left(t+2l\right),\ldots ,x\left(t+ml\right)\right] \end{array}$$

where *x*(*t*) could be displacement and velocity of segment centers of mass (CoM) or joint angles and angular velocities. The time delay *l* of the movement kinematics is set such that the state space coordinates are uncorrelated and dimension *m* is set such that the trajectories in the state space do not overlap. The first local minimum of the average mutual information function is often considered as time delay *l* and the minimum number of false nearest neighbors is used to find the correct number *m* of delays (Abarbanel, 1996; Kantz and Schreiber, 2004).

**Figure 1:**
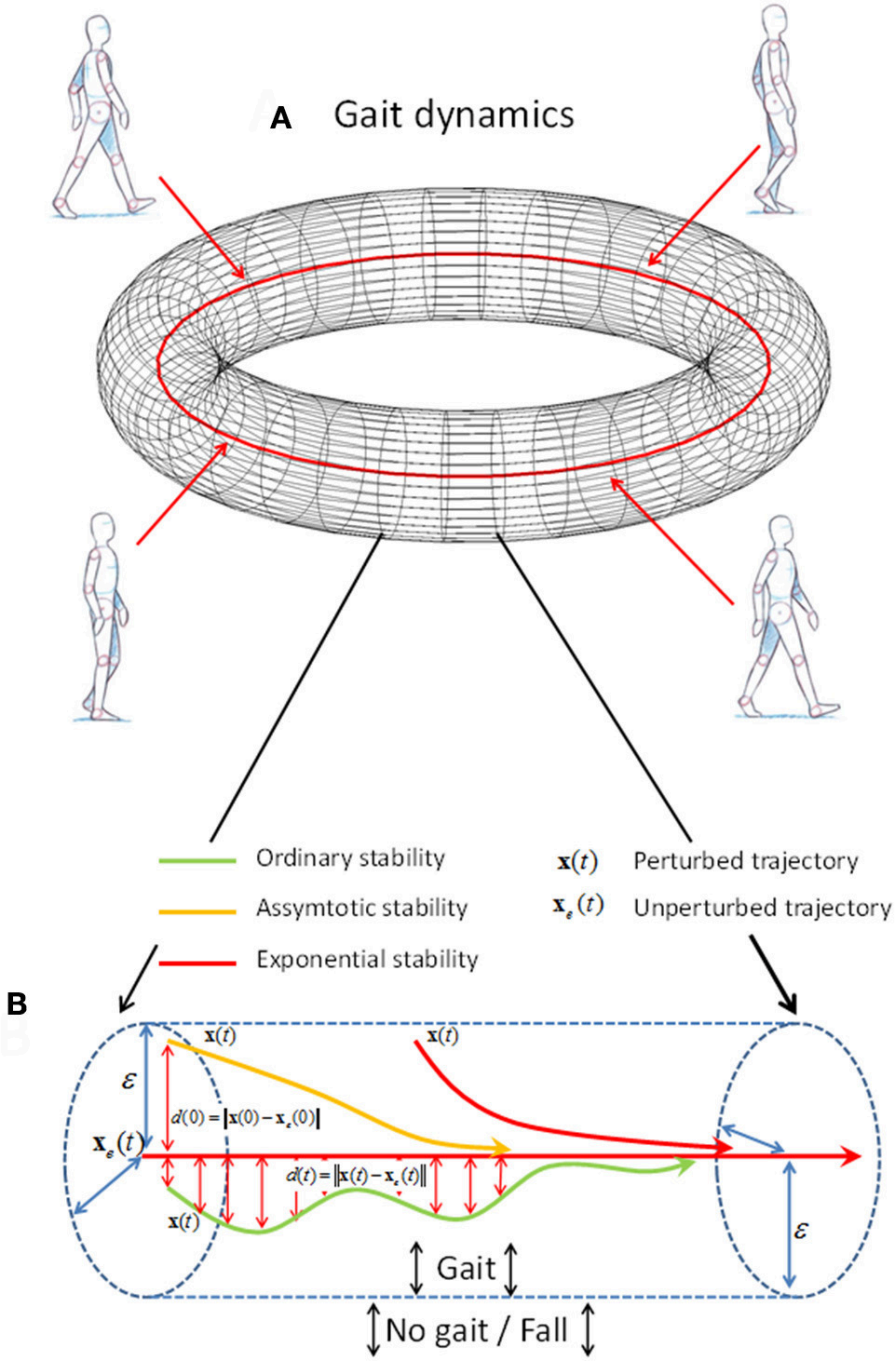
A schematic representation of the construct of stability of motion. **(A)** The unperturbed gait dynamics **x**_*e*_(*t*) is represented as a red orbit with a ε radius torus around. The ε-boundary represents a small distance for which the perturbed gait dynamics **x**(*t*) for a small perturbation of size *d*(0) < δ remain within. **(B)** A schematic representation of ordinary stability (*green line*), asymptotic stability (*yellow line*), and exponential stability (*red line*) within a section of the torus (*blue dashed lines*). The distance *d*(*t*) between the unperturbed **x**_*e*_(*t*) and the perturbed gait dynamics **x**(*t*) has to be within the ε < radius for the dynamics to be stable. In addition, the yellow arrow has to approach the unperturbed trajectory **x**_*e*_(*t*) (*red center line*) as *t* → ∞ when the gait dynamics is asymptotic stable. Moreover, the red arrow has to approach the red arrow exponential fast where *d*(*t*) ≤ *d*(0)exp(λ*t*) and the Lyapunov exponent λ < 0, when the dynamics is exponentially stable.

Lyapunov's theory of dynamic stability indicates *how* the gait dynamics **x**(*t*) react to a perturbation (Lyapunov, 1992). The size of a perturbation is assessed as the distance *d*(0) = ||**x**(0) – **x**_*e*_(0)|| between the perturbed point **x**(0) and unperturbed point **x**_*e*_(0) in the reconstructed state space (see upper red vertical arrow in Figure 1B). The reaction to the perturbation is numerically defined as the temporal change, *d*(*t*) = ||**x**(*t*) – **x**_*e*_(*t*)||, of the initial distance *d*(0) between the perturbed gait trajectory **x**(*t*) and the unperturbed gait trajectory **x**_*e*_(*t*). The theory of dynamic stability defines the three constructs according to the temporal change in *d*(*t*); ordinary stability, asymptotic stability, and exponential stability (see Figure 1).

**Figure 2:**
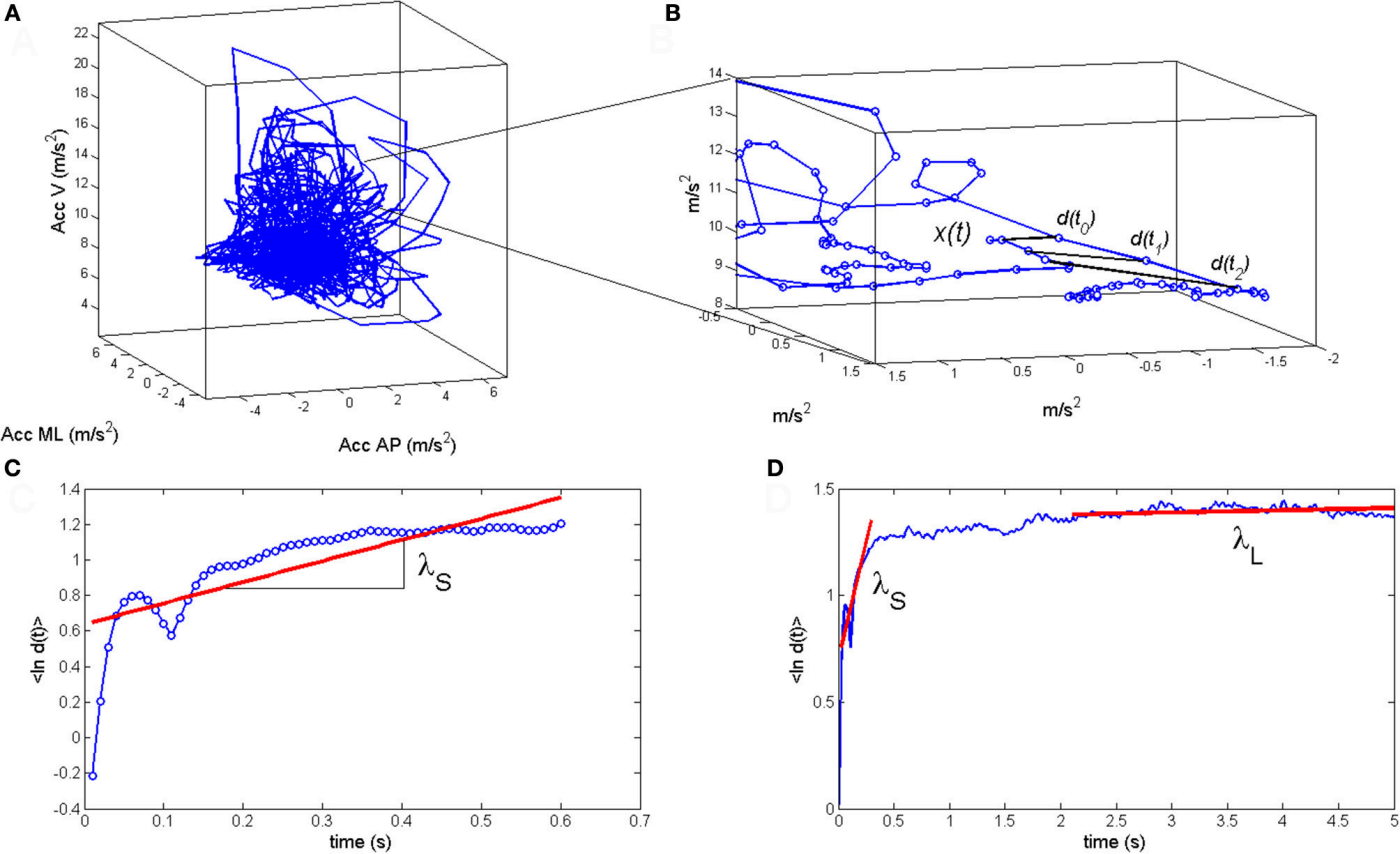
**(A)** Example of a reconstructed state space of the anterioposterior, mediolateral, and vertical trunk acceleration during daily life walking. **(B)** The reaction size *d*(*t*) is estimated as the distance between the two nearest neighbor trajectories in the reconstructed state space. **(C)** The maximum finite size Lyapunov exponent λ_*S*_ is estimated as the linear regression slope of 〈ln *d*(*t*)〉 where the brackets indicates the mean of logarithm of *d*(*t*) across all points in the reconstructed state space. **(D)** The short-range exponent λ_*S*_ is estimated for the range of one step to one stride and the long range exponent λ_*L*_ is estimated 4–10 steps.

Exponential stability is by far the most used stability construct in modeling and signal processing in general and in gait analysis in particular, because it provides a measure, called Lyapunov exponent, to parameterize *d*(*t*) (Dingwell and Cusumano, 2000; Bruijn et al., 2013; van Schooten et al., 2013). The exponential stability, *d*(*t*) = *d*(0)exp(λ*t*), can be estimated as the linear regression slope, λ, of the log(*d*(*t*))-curve in each direction of the reconstructed state space. The exponent λ_*i*_ is most reliably estimated for the most unstable direction of the reconstructed state space, where it is referred to as the *maximum finite size Lyapunov exponent* (Rosenstein et al., 1993; see Figure 2). Furthermore, in gait analysis, the short-range exponent λ_*S*_ computed across a single step or stride is considered to be most representative for dynamic stability of human gait (van Schooten et al., 2011; Bruijn et al., 2013; see Figures 2C,D). The gait dynamics are referred to as *local dynamically stable* or *unstable* when λ_*S*_ < 0 or λ_*S*_ > 0, respectively, indicating that the average distance *d*(*t*) between the perturbed trajectory **x**(*t*) and the unperturbed trajectory **x**_*e*_(*t*) decreases or increases exponentially.

Several reports suggest that λ_*S*_ is one of the most valid measures of gait stability (cf. Bruijn et al., 2013). To the authors' knowledge, all studies have reported λ_*S*_ > 0 for gait dynamics, irrespective of the measurement device and patient group. λ_*S*_ has shown to predict the risk of falling in gait models (Su and Dingwell, 2007; Kurz et al., 2010; Roos and Dingwell, 2011; Bruijn et al., 2012), discriminate between fallers and non-fallers in an older population (Lockhart and Liu, 2008; Toebes et al., 2012), and reflect the reaction to experimentally induced perturbations (Chang et al., 2010; McAndrew et al., 2011; Sloot et al., 2011; Hak et al., 2012).

### Fractional stability and phase transitions in human gait

**Figure 3:**
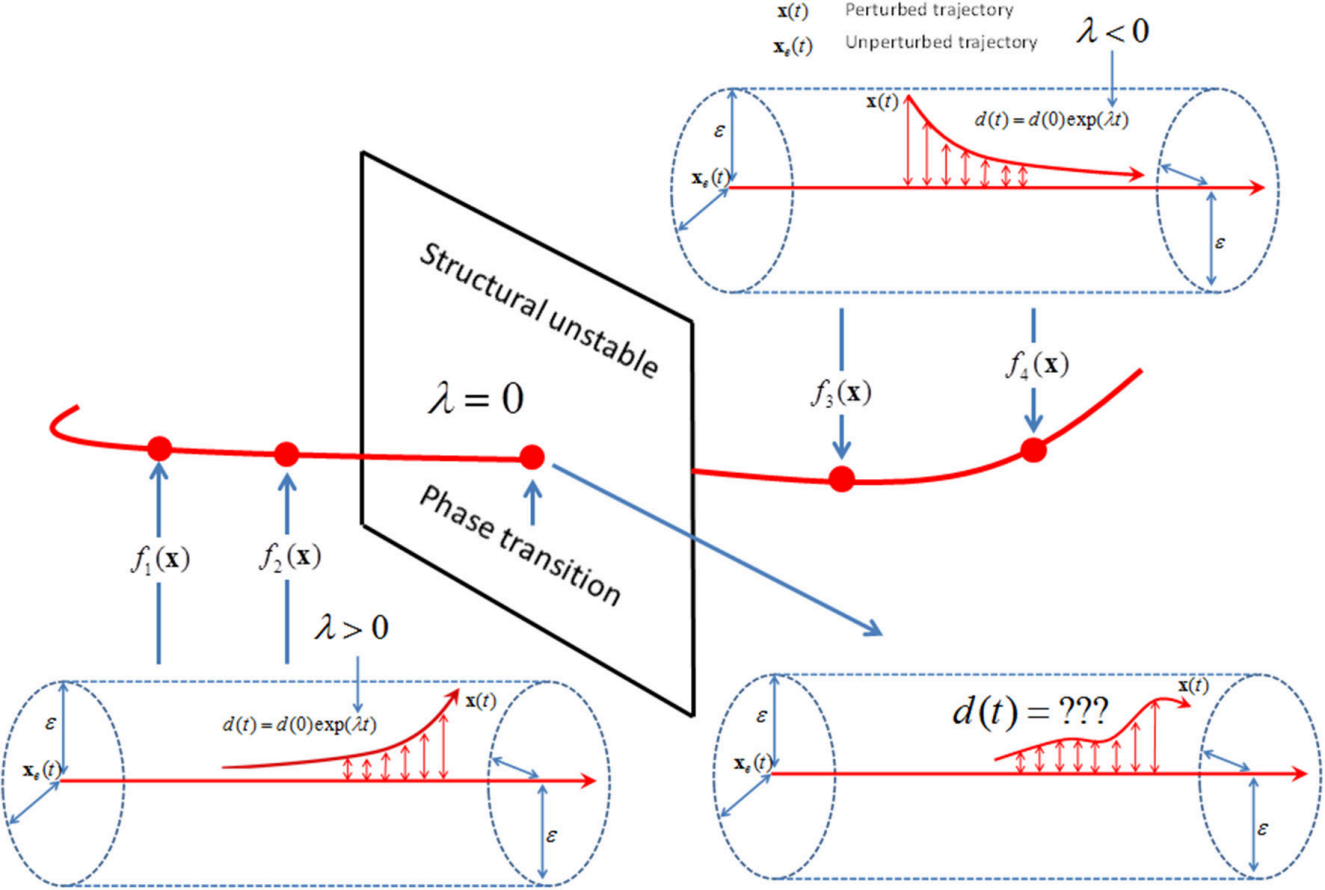
Schematic illustration of a trajectory (*red line*) in function space where each point on the trajectory represents a specific equation of motion *f* (*x*). The gait dynamics is structural stable when the stability properties (red arrow within the ε-radius) does not change by changes from *f*_1_(*x*) to *f*_2_(*x*) or from *f*_3_(*x*) to *f*_4_(*x*). The gait dynamics *f*_1_(*x*) and *f*_2_(*x*) as well as *f*_3_(*x*) and *f*_4_(*x*) is structural equivalent with the same set of positive and negative λ. However, the gait dynamics is structural unstable when the unstable dynamics (i.e., λ > 0) of *f*_2_(*x*) changes to stable dynamics (i.e., λ < 0) of *f*_3_(*x*). In the close vicinity of the critical point of phase transition (*red point on the two-dimensional intersection*), the stability property of the gait dynamics *f* (*x*) is no longer defined by the exponential *d*(*t*) = *d*(0)exp(λ*t*).

Exponential stability λ_*S*_ of the gait kinematics will only define the degree of dynamic stability when no phase transitions are present in the gait dynamics. The human gait cycles involve multiple phases; a single support phase initiated by a push-off where the body's center of mass (CoM) moves as an inverted pendulum, and a double support phase initiated by a heel-strike which decelerates the body's CoM displacement in the propulsion direction. Even very simple biomechanical models have to include transformations of the equation of motion between the single and double support phase, thereby changing dynamic stability properties (Norris et al., 2008; Srinivasan et al., 2008; Huang et al., 2012). At the transformation, reaction size *d*(*t*) is a non-exponential function of time and cannot be estimated by an exponential function (Andronov and Pontrjagin, 1937; see intersection in Figure 3). It has been hypothesized that the human neuromuscular system remains in the close vicinity of the critical points of phase transitions where the system is highly adaptable to changing influx of energy, information, and matter (Haken, 1977, 1983; Bak et al., 1988; Kelso, 1995).

**Figure 4:**
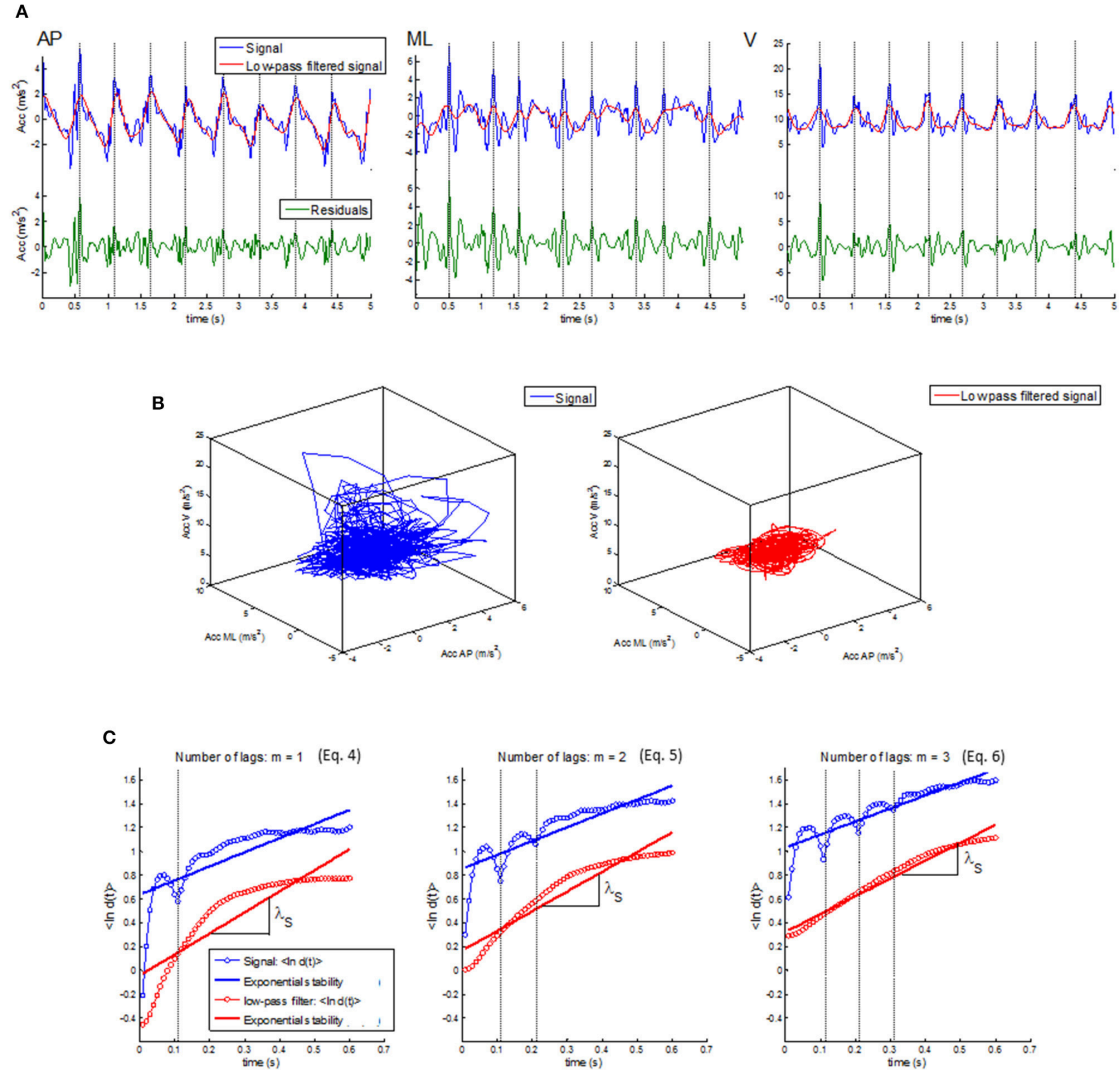
A representative example of the influence of within-step transitions in the gait dynamics on the non-exponential singularities in the reaction size *d*(*t*). **(A)** The trunk acceleration signal (*blue trace*) and low-pass filtered signal (*red trace*) for the anterioposterior (AP; *upper left*), mediolateral (ML; *upper middle*), and vertical direction (V; *upper right*). The green traces in the lower panels shows the corresponding high frequency residuals with step-to-step modulations generated by heel-strikes and push-offs (*dashed vertical lines*). **(B)** The reconstructed state space including AP, ML, and V direction for the trunk acceleration (blue trace in left) and the low frequency filtered signal (red trace in right). **(C)** The mean reaction distance, 〈ln *d*(*t*)〉, and the regression line, *d*(*t*) = *d*(0)exp(λ*t*), for the trunk acceleration (*blue dots and line*) and the low-pass filtered signal (*red dots and line*) for reconstructed state space by Equations (4–6) for *m* = 1, 2, and 3 lags, respectively. The singularities in 〈ln *d*(*t*)〉 vanish in the low-pass filtered signal when the within-step transitions are removed. Notice that the singularity for the trunk acceleration for each of the *m* lags appears at time *t* = *ml*/Δ*t* where *l* is the lag size of the state space reconstruction and Δ*t* is the sampling frequency.

Several reports indicate that the distance *d*(*t*) assessed for the computation of exponential stability has a non-exponential shape (e.g., Figure 1 in Bruijn et al., 2009; Figure 7 in Dingwell and Cusumano, 2000; Figure 5 in Dingwell and Marin, 2006; Figure 2 in Lockhart and Liu, 2008; Figure 1 in Sloot et al., 2011). Singularities appear in the reaction distance *d*(*t*) at *t* = *l*, 2*l*, …, *ml* where *l* is the lag size and *m* is the number of lags chosen for the state space reconstruction according to Equation (1) (van Schooten et al., 2013). In Figure 4, we show an example of reaction distance *d*(*t*) for unfiltered and 3 Hz low-pass filtered trunk acceleration signals of a community-dwelling older adult during daily life walking. The singularities in *d*(*t*) vanish with low-pass filtering for cut-offs less than 3 Hz, which is close to the 2 Hz frequency of the step cycle. Thus, the singularities in *d*(*t*) seem to be generated by the high frequency, intra-stride, details around the periodic low frequency gait cycle and not by noise-related errors in the signal as previously suggested (van Schooten et al., 2013). Figure 4 indicates that exponential stability, *d*(*t*) = *d*(0)exp(λ*t*), may not accurately fit these high frequency transitions in the acceleration signal generated by intra-step events, like push-off and heel strike, or transitions in the walking circumstances, like turning corners, walking stairs or doing additional tasks while walking.

Fractional calculus has been suggested as a mathematical tool to define equations of motion of structurally unstable dynamics with phase transitions (see Figure 3; Hilfer, 1995; Podlubny, 1998, 2002; Zaslavsky, 2005; West, 2006). In fractional calculus, the Newtonian operators, *d*/*dt* and *d*/*d***x**, are special cases of the fractional order operators, *d*^α^/*dt*^α^ and *d*^β^/*d***x**^β^ for α = 1 and β = 1. The fractional indices, α and β, of the differential operators are considered to describe the universal class of the dynamics in the presence of phase transition (Zaslavsky, 2005). Assuming that the unknown gait dynamics can be described by an equation of motion with fractional order operators, *d*^α^/*dt*^α^ and *d*^β^/*d***x**^β^, the construct of exponential stability, *d*(*t*) = *d*(0)exp(λ*t*) can be extended by the following construct of fractional stability introduced by Yu et al. (2013):

(2) $$\begin{array}{r} d\left(t\right)\leq Ct^{\beta -1}E_{\alpha ,\beta }\left(\lambda_{f}t^{\alpha }\right) \end{array}$$

where $E_{\alpha ,\beta }\left(\lambda_{f}t^{\alpha }\right)$ is the generalized Mittag-Leffler function which is the generalization of an exponential function defined by the following equation:

(3) $$\begin{array}{r} E_{\alpha ,\beta }\left(\lambda_{f}t^{\alpha }\right)=\overset{\infty }{\underset{k=0}{\sum }}\frac{{\left(\lambda_{f}t^{\alpha }\right)}^{k}}{\Gamma \left(k\alpha +\beta \right)} \end{array}$$

**Figure 5:**
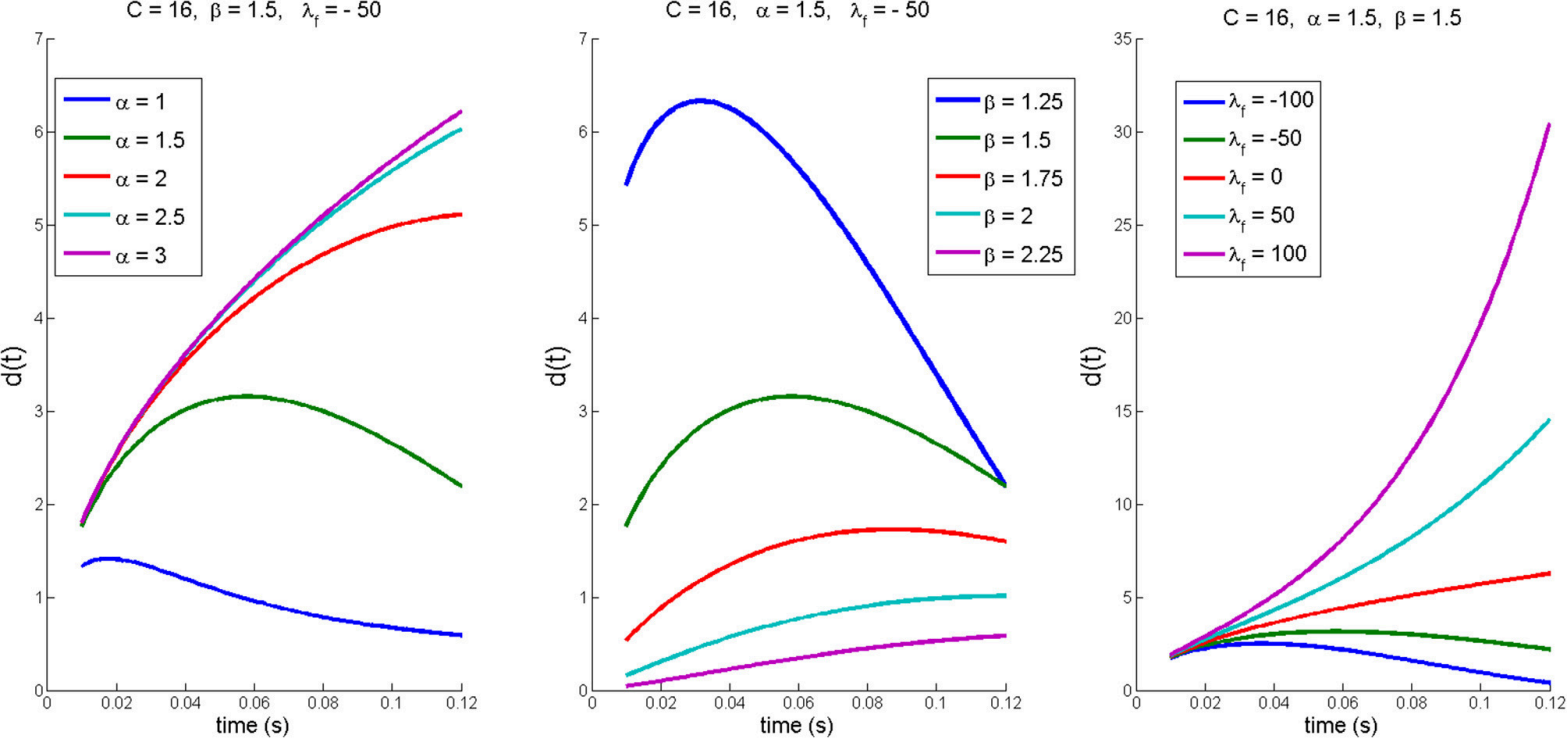
Illustration of non-exponential shapes of reaction size *d*(*t*) defined by fractional stability (see Equation 2). (***Left***) Changes in *d*(*t*) when fractional order α is varied and β = 1.5 λ_*f*_ = –50, and *C* = 16 are fixed. (***Middle***) Changes in *d*(*t*) when fractional order β is varied and α = 1.5, λ_*f*_ = −50, and *C* = 16 are fixed. (***Right***) Changes in *d*(*t*) when fractional Lyapunov exponent is varied and α = 1.5, β = 1.5, and *C* = 16 are fixed.

The stability of the dynamics is quantified by the fractional Lyapunov exponent, λ_*f*_, which has to be considered in relation to the fractional indices, α and β, of the differential operator. A structurally unstable system is fractionally stable when λ_*f*_ < 0, or fractionally unstable when λ_*f*_ > 0. The left and middle panels in Figure 5 show that reaction size *d*(*t*) are scaled along the time and space dimension by α and β leading to different non-exponential shapes of *d*(*t*) for the same λ_*f*_. Thus, in contrast to exponential stability, the reaction size *d*(*t*) defined by Equation (2) may change non-monotonically depending on λ_*f*_ in combination with α and β (see Figure 5). The additional advantage of fractional stability (i.e., Equation 2) is that it is a general stability construct that contains exponential stability, *d*(*t*) = *d*(0)exp(λ*t*), as a special case when α = 1 and β = 1 where the generalized Mittag-Leffler function (i.e., Equation 3) reduces to the exponential function, *t*^β^ ^−1^ = 1, and *C* = *d*(0).

### Fractional stability in trunk acceleration dynamics of community dwelling older persons: a proof-of-concept study

#### Participants and measurement device

Inertial sensor data recorded during daily-life walking in 172 community-dwelling older adults (the FARAO study; van Schooten et al., 2011) were re-analyzed. A summary of the participant characteristics is provided in Table 1. Participants wore a small inertial sensor (DynaPort Hybrid, McRoberts, The Hague, Netherlands; 87 × 45 × 14 mm, 74 g) with a belt over the lower back during 1 week in daily life. The inertial sensor sampled 3D accelerations at 100 samples/s and has range of ±6 g and resolution of ±1 mg. Participants were instructed to wear the inertial sensor at all times, except during aquatic activities which may damage the device. The original study was approved by the medical ethics committee of the VU Medical Center (protocol 2010/290) and all participants had provided written informed consent to partake in the study.

**Table 1:** Demographic variables and clinical tests of the community-dwelling older persons.

**Community-dwelling older adults (*****N*** = **172)**	
Gender (% female)	50
Age (yrs, mean ± SD)	75.7 ± 6.7
Height (cm, mean ± SD)	171.0 ± 8.5
Weight (kg, mean ± SD)	73.7 ± 11.8
Assisted living (%)	5.8
Residential care (%)	1.7
Walking aid (%)	17.4
MMSE (median/range)	28/10
≥1 falls in past 6 months (%)	41.9

#### Pre-processing of data

The following procedure was used to identify daily-life walking: First, the 3D acceleration signals for walking bouts with ≥ 3 s duration were identified by the McRoberts activity detection algorithm (McRoberts bv, the Hague, the Netherlands). Second, the 3D acceleration signals for walking bouts with duration of ≥30 s were extracted to provide a sufficient number of steps in each bout for the computation of gait stability. Third, all included walking bouts were converted into equal sized 30-s epochs (i.e., 3,000 samples) to provide a consistent sample size for the computation of the gait stability measures. Fourth, all included epochs were visually checked and non-walking activity was excluded based on lack of periodicity of the trunk acceleration in the vertical and anterioposterior direction. A total of 42,431 walking epochs were identified across 172 older adults (median number of epochs: 210.5, range: 21 to 906).

#### Computation of fractional stability

The trunk acceleration dynamics was reconstructed by the following equation:

(4) $$\begin{array}{r} \text{x}\left(t\right)=\left[x_{AP}\left(t\right),x_{AP}\left(t+l\right),x_{ML}\left(t\right),x_{ML}\left(t+l\right),x_{V}\left(t\right),x_{V}\left(t+l\right)\right] \end{array}$$

where lag size *l* is the mean of the first minimum of the average mutual information function across the AP, ML, and V direction. The minimum percentage of false nearest neighbors was obtained for a single lag. The reaction size *d*(*t*) to initial perturbation *d*(0) was assessed as an average across all state space points **x**(*t*) following the method introduced by Rosenstein et al. (1993). Fractional stability was estimated for a short range of *d*(*t*) between the initial perturbation and the first singularity. Equation (2) was fitted to a five-time up-sampled version of *d*(*t*) in this range using a non-linear least square optimization procedure. We used the lsqcurvefit function in Matlab for this procedure, where α and β parameters were assumed to be in the interval [0, 5] and [0, 3], respectively, with *C* > 0 and no bounds assumed for λ_*f*_. Initial conditions α = 2, β = 2, λ_*f*_ = 1 and *C* = *d*(0) were set for optimization but all optimizations converged to the same minima irrespective of initial condition and upper boundary conditions of α and β. We also tested the mlffit2 script released by Podlubny et al. (2012) and it produced similar results.

The present study also included double (Equation 5) and triple lags (Equation 6) to investigate the relationship between fractional stability metrics, α, β, and λ_*f*_ of the first singularity in *d*(*t*) of different number of lags:

(5) $$\begin{array}{r} \text{x}\left(t\right)=\left[\begin{array}{c} x_{AP}\left(t\right),x_{AP}\left(t+l\right),x_{AP}\left(t+2l\right),x_{ML}\left(t\right),x_{ML}\left(t+l\right),\\ x_{ML}\left(t+2l\right),x_{V}\left(t\right),x_{V}\left(t+l\right),x_{V}\left(t+2l\right) \end{array}\right] \end{array}$$

(6) $$\begin{array}{r} \text{x}\left(t\right)=\left[\begin{array}{c} x_{AP}\left(t\right),x_{AP}\left(t+l\right),x_{AP}\left(t+2l\right),x_{AP}\left(t+3l\right),\\ x_{ML}\left(t\right),x_{ML}\left(t+l\right),x_{ML}\left(t+2l\right),x_{ML}\left(t+3l\right),\\ x_{V}\left(t\right),x_{V}\left(t+l\right),x_{V}\left(t+2l\right),x_{V}\left(t+3l\right) \end{array}\right] \end{array}$$

The state space reconstructions of Equation (5) and Equation (6) are included in the figures in the results section Results below to illustrate how *d*(*t*) changes with the number of lags.

#### Surrogate test for phase transitions

**Figure 6:**
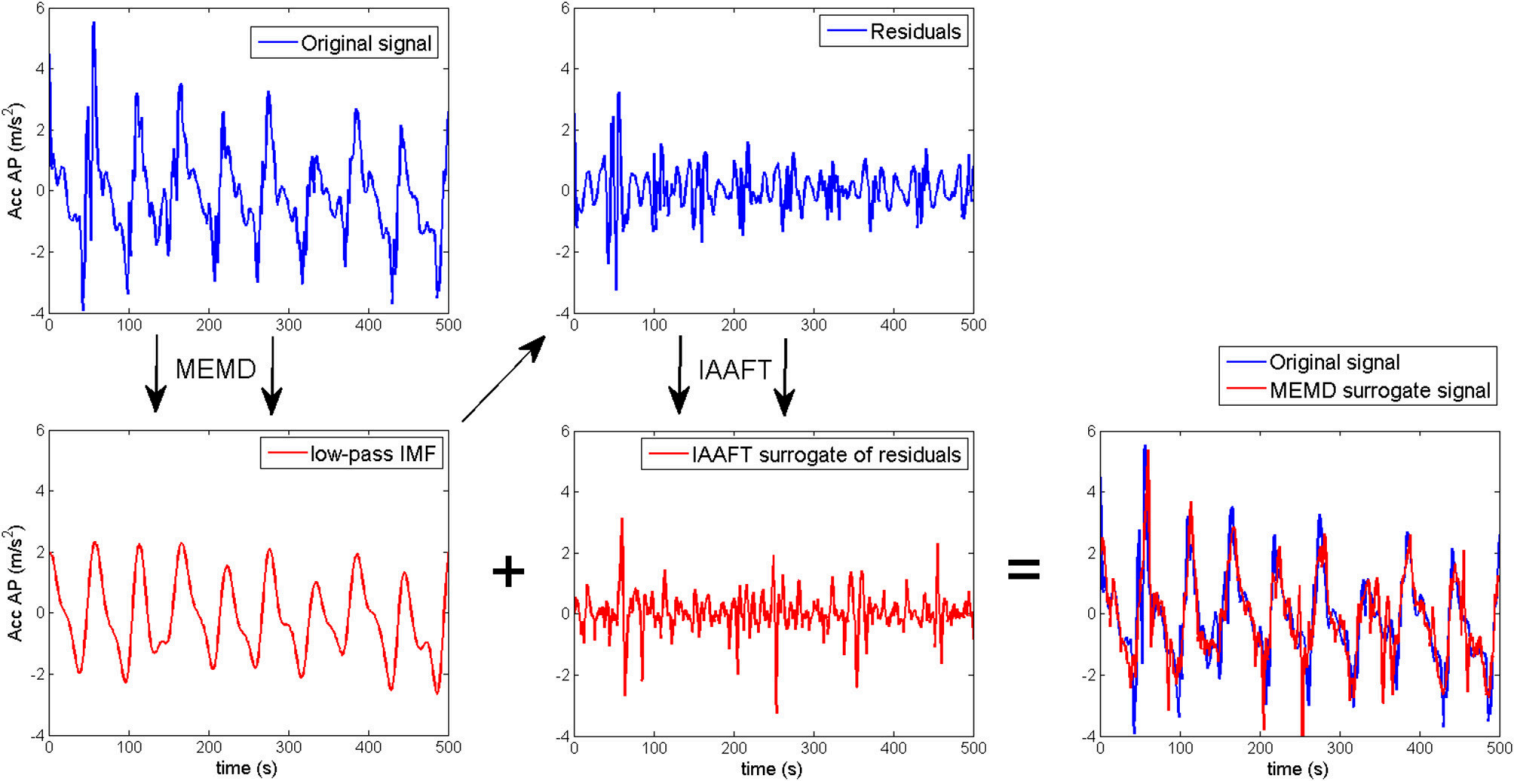
Schematic representation of the generation of multivariate empirical mode decomposition (MEMD) surrogates. MEMD splits the acceleration signals into a low frequency periodic motion (red trace) and high frequency intra-step motion (blue trace). The phase-dependent modulation of the high frequency motion is removed in iterated amplitude adjusted fourier transform (IAAFT) surrogates (red trace) whereas a the distribution and power spectral density remain unaltered. Finally, MEMD surrogates are composed as the sum of high frequency IAAFT surrogates (red trace) and low frequency periodic motion assessed by the MEMD.

A surrogate test based on multivariate empirical mode decomposition (MEMD) was used to assess the influence of phase-dependent changes of the trunk acceleration dynamics on the estimated fractional stability parameters α, β, and λ_*f*_. MEMD defines the components of the gait dynamics, called intrinsic mode functions (IMF), in an iterative way from high to low frequency modes, where the frequency range is dependent on intrinsic properties of the dynamics (Rehman and Mandic, 2010). Thus, IMFs are more related to the process measured than conventional Fourier functions for predefined frequency ranges. In contrast to univariate EMD, MEMD are able to detect common spectral modalities across all dimensions of the reconstructed gait dynamics. The sum of intrinsic mode functions assessed by MEMD for level 5 and above reconstructed the main periodicity of the gait dynamics, and the sum of IMFs for level 1 to 4 reconstructed the high frequency intra-step details of the gait dynamics (see Figure 6). Iterated amplitude adjusted Fourier transform (IAAFT) was used to generate surrogates for the high frequency details that were subsequently added to the main periodicity of the gait dynamics (i.e., IMF for level 5 and above). IAAFT preserves the distribution and the power spectral density of the high frequency details of the original dynamics, while the phasic behavior of these details is removed (Schreiber and Schmitz, 1996). Thus, this surrogate procedure can test the influence of intra-step phase-dependent changes in the trunk acceleration dynamics on the fractional stability. A MEMD surrogate was created for the dynamics of each walking epoch. Further technical details for the MEMD surrogate test are provided in Appendix A.

#### Statistics

The median of fractional stability parameters, λ_*f*_, α, β, and *C*, for Equation (2) were computed across all 30-s epochs for each person. In addition, the conventional short-term Lyapunov exponent, λ_*S*_, was assessed for the same *d*(*t*) using Rosenstein et al.'s (1993) method. The goodness of fit of fractional stability (Equation 2) was compared to exponential stability by the AICc criterion, which penalizes the goodness of fit of fractional stability according to the additional number of parameters in Equation 2. The differences in the goodness of fit were statistically tested by the relative likelihood, RL = exp([AICc_1_–AICc_2_]/2) (Burnham et al., 2011) and fractional stability was considered as a superior model when RL < 0.05. Pearson correlation with Bonferroni correction for multiple comparisons were used to assess the relationship between fractional stability, as median values of λ_*f*_, α, β, and exponential stability, as median values of λ_*S*_. Furthermore, Pearson correlations of fractional stability metrics, *alpha*, β, and λ_*f*_ between different number of lags (i.e., Equations 4–6) were calculated to assess the consistency of α, β, and λ_*f*_ across changes in the state space reconstruction method. The difference between λ_*f*_, α, and β, of the original dynamics and $\lambda_{f}^{surr},\alpha^{surr},\beta^{surr}$ of the MEMD surrogate dynamics were tested with paired samples *t*-tests.

## Results

**Figure 7:**
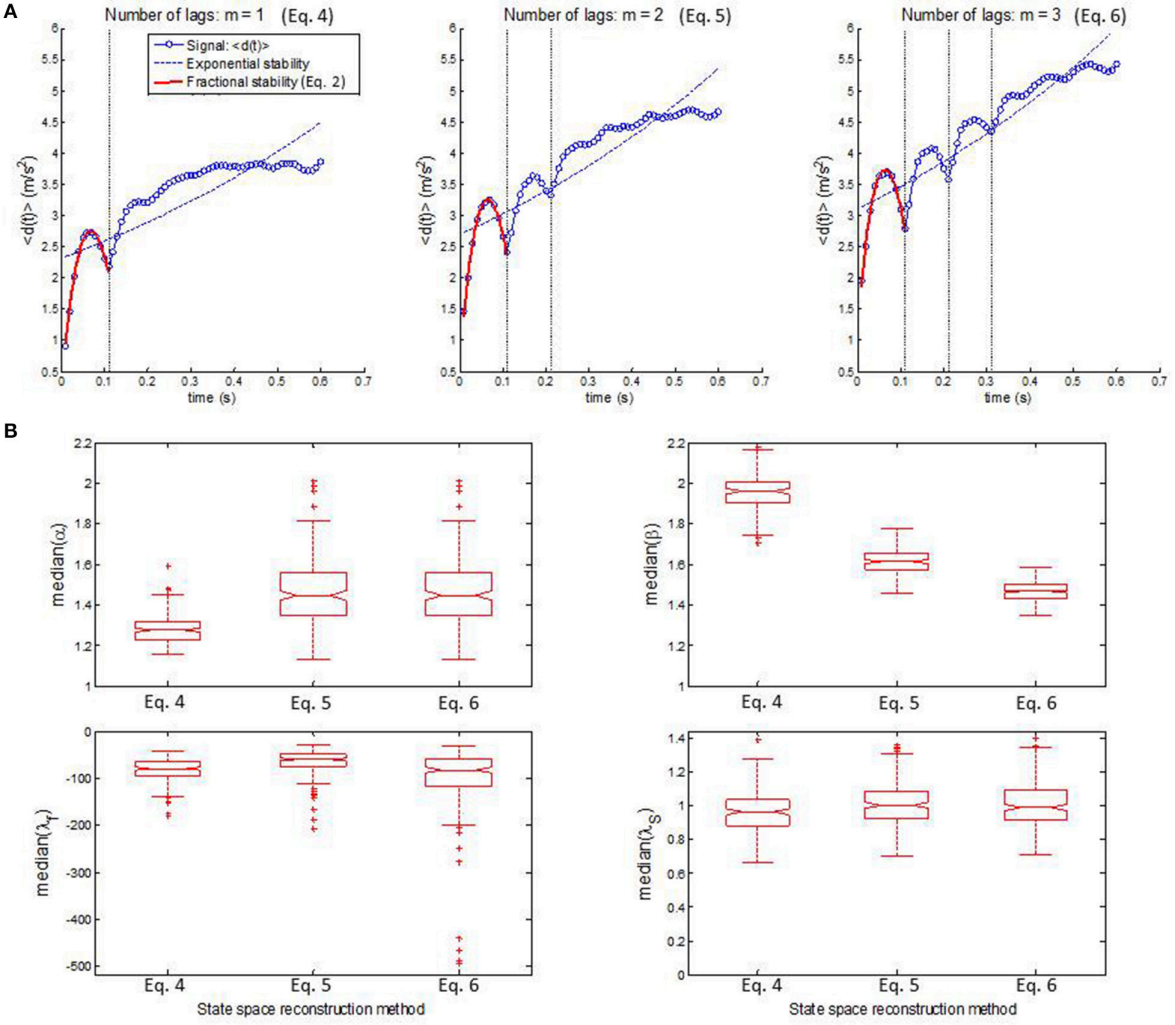
**(A)** Representative example of fractional stability in Equation (2) (*bold red lines*) fitted to the *d*(*t*) (*blue dots*) for state space reconstruction in Equation (4) (*left*), Equation (5) (*middle*), and Equation (6) (*right*). Fractional stability in Equation (2) represented a considerable improved model of *d*(*t*) compared to exponential stability *d*(*t*) = *d*(0)exp(λ_*S*_*t*) (*dashed blue lines*) for all state space reconstruction methods. **(B)** Boxplots of the median values of the fractional stability parameters, α (*upper left*), β (*upper right*), and λ_*f*_ (*lower left*) together with median values of exponential stability λ_*S*_ (*lower right*). Note that the center of the box represents the median and the upper and lower borders of the box represent the 75th and 25th percentile, respectively. The whiskers represent the most deviating values within 1.5 times the interquartile range from the median value whereas values outside this range are represented as outliers.

**Figure 8:**
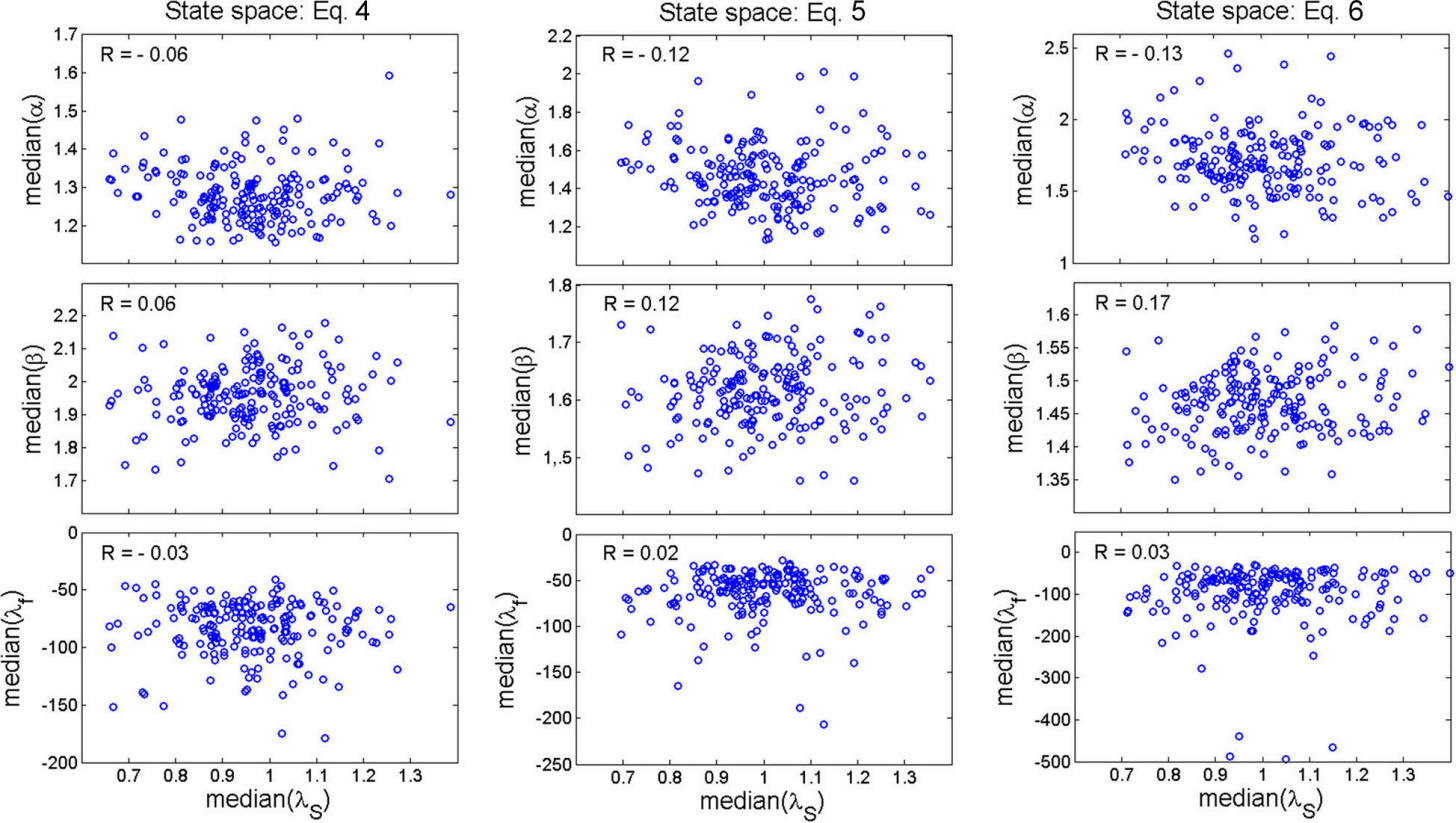
Scatterplot of the relationship between the median values of the fractional stability parameters, α, (***upper*** *row of panels*), β (***middle*** *row of panels*), λ_*f*_ (***lower*** *row of panels*), and the median values of the exponential stability parameter λ_*S*_ for state space reconstruction method of Equation (4) (***left*** *column of panels*), Equation (5) (***middle*** *column of panels*), and Equation (6) (***right*** *column of panels*). The scatterplots are shown with corresponding Pearson correlation coefficients.

Figure 7 shows fractional stability (red line) and exponential stability (dashed blue line) fitted to a representative example of distance *d*(*t*). The goodness-of-fit statistics (RL < 0.00001) was in favor of fractional stability for all walking epochs, which indicates that fractional stability provided a considerably improved fit compared to exponential stability. All walking epochs had a negative λ_*f*_ (i.e., λ_*f*_ ϵ [−1094.5, −8.7]) indicating that the gait dynamics were fractionally stable (see median λ_*f*_ in Figure 7B). The finding of fractionally stable gait was in contrast with the positive λ_*S*_, which indicate exponential instability of gait dynamics for all epochs (λ_*S*_ ϵ [0.41, 2.10]). The operator α and β had a range of α ϵ [0.91, 3.79] and β ϵ [1.23, 2.42], while median values for all participants were larger than the special case of exponential stability, α = 1 and β = 1 (see Figure 7B). In addition, no significant correlation (*R* < 0.17) was found between exponential stability λ_*S*_ and fractional stability parameters (i.e., α, β, and λ_*f*_ in Equation 2) as shown in Figure 8. A highly significant correlation (R > 0.83, *p* < 0.0001) was found between the fractional stability parameters of the different state space reconstruction methods (i.e., Equations 4–6) indicating robustness of the parameters to the choice of state space reconstruction method.

**Figure 9:**
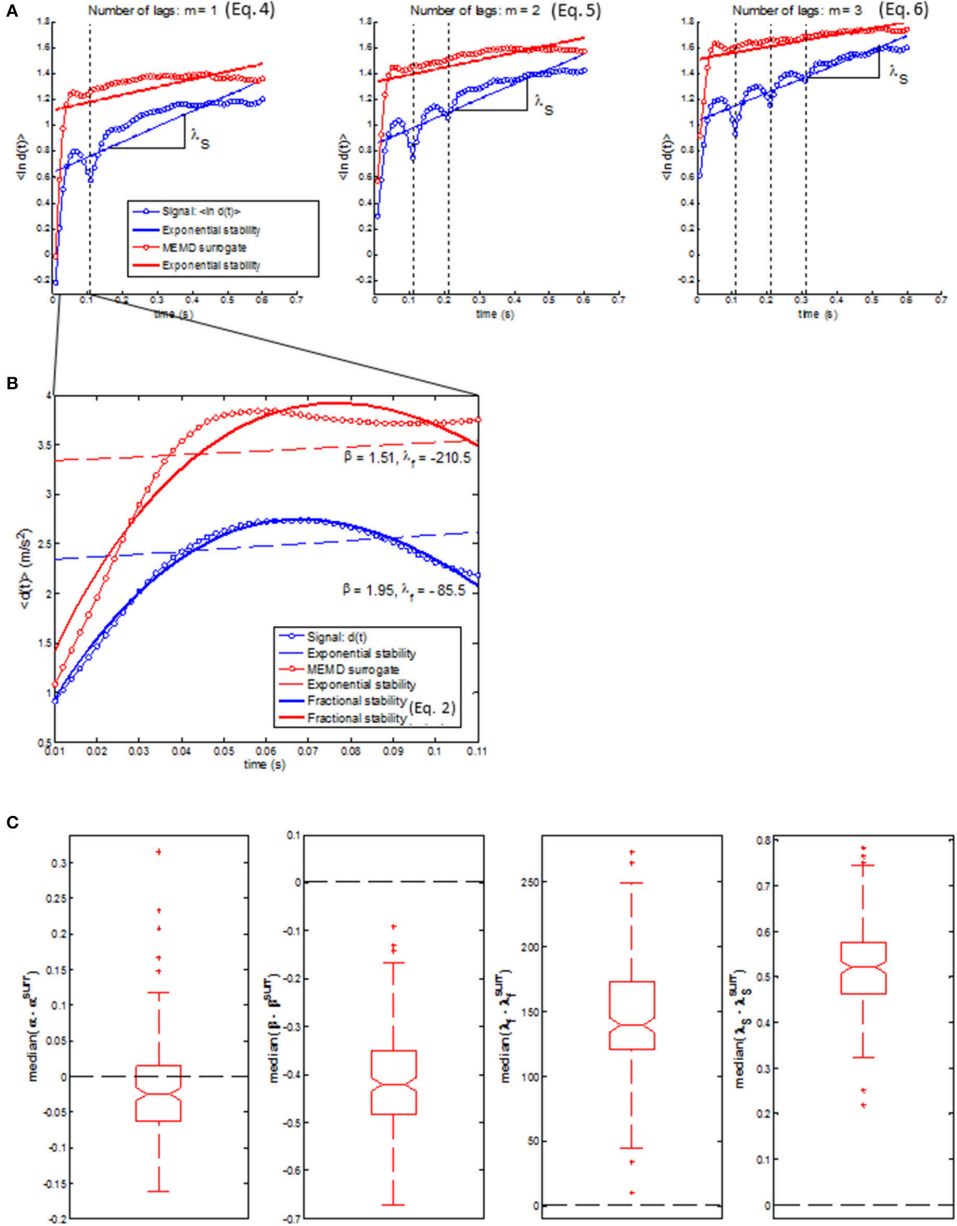
**(A)** A representative example of the difference between *d*(*t*) of the original trunk acceleration (blue trace) and *d*(*t*) of the MEMD surrogates (red trace) where the regression line used to calculate λ_*S*_ is a bold blue and red line, respectively. **(B)** The up-sampled *d*(*t*) from initial perturbation *d*(0) to the first singularity of the original trunk acceleration (blue trace) and *d*(*t*) of the MEMD surrogates (red trace) where the nonlinear least square fit of Equation (2) is shown as a bold blue and red curve, respectively. **(C)** Boxplots of the median difference between fractional stability of the original trunk acceleration and the MEMD surrogates; median (α−α^*surr*^) (*left*), median (β−β^*surr*^) (*middle left*), median $\left(\lambda_{f}-\lambda_{f}^{surr}\right)$ (*middle right*), and median difference between exponential stability of the original trunk acceleration and the MEMD surrogates; median $\left(\lambda_{S}-\lambda_{S}^{surr}\right)$. Note that the center of the box represents the median and the upper and lower borders of the box represent the 75th and 25th percentile, respectively. The whiskers represent the most deviating values within 1.5 times the interquartile range from the median value whereas values outside this range are represented as outliers.

Figure 9 shows that the singularity in *d*(*t*) vanished for the MEMD surrogates. This indicates that the phase-dependent high frequency modulations in the trunk acceleration, probably related to push-off and heel-strike events, are the origin of the singularities in *d*(*t*). Compared to actual data, the MEMD surrogates had significantly more negative values of λ_*f*_ and larger values of β which, in combination, led to a smaller decrease of *d*(*t*) toward the first singularity (see Figure 9B). These findings, based on fractional stability, suggest that the gait dynamics destabilizes when the phase-dependent modulation of trunk accelerations is removed. In contrast to fractional stability, the conventional short-term Lyapunov exponent λ_*S*_ decreased for the MEMD surrogates indicating more stable dynamics based on exponential stability (see Figure 9A).

## Discussion

The present paper proposes the application of the concept of fractional stability to the analysis of gait. Fractional stability is able to quantify stability in the presence of intra-step phase transitions. The application of fractional stability to trunk acceleration data of daily-life walking in community-dwelling older adults showed that fractional stability was a better model of *d*(*t*) compared to exponential stability for all walking epochs. Interestingly, fractional stability indicated that gait dynamics are stable. This result is in contrast to exponential stability, which in earlier studies indicated that gait dynamics are unstable (Dingwell and Cusumano, 2000; Toebes et al., 2012; Bruijn et al., 2013; Terrier and Reynard, 2015; Hamacher et al., 2016; van Schooten et al., 2016; de Melker Worms et al., 2017).

Ever since Dingwell and Cusumano (2000) introduced exponential stability to gait analysis, this stability construct has been applied to a wide variety of walking conditions and populations. The short-term Lyapunov exponent, λ_*S*_, was shown to distinguish well between elderly fallers and non-fallers, to be sensitive to experimentally induced balance perturbations, and to describe the stability of simple models of human walking well (Lockhart and Liu, 2008; Kurz et al., 2010; Roos and Dingwell, 2011; Sloot et al., 2011; van Schooten et al., 2011; Toebes et al., 2012; Bruijn et al., 2013; Terrier and Reynard, 2015; de Melker Worms et al., 2017). However, the construct of exponential stability is not applicable in the presence of phase transitions as detailed by Section “Fractional stability in trunk acceleration dynamics of community dwelling older persons: A proof-of-concept study”. The MEMD surrogate tests indicated that *d*(*t*) is sensitive to phasic intra-step changes of trunk acceleration, likely due to heel-strikes and toe-offs. The surrogate tests further showed that these phasic changes generated the singularity in *d*(*t*) that was shown in several previous studies of gait stability (e.g., see Figure 1 in Bruijn et al., 2009; Figure 7 in Dingwell and Cusumano, 2000; Figure 5 in Dingwell and Marin, 2006; Figure 2 in Lockhart and Liu, 2008; Figure 1 in Sloot et al., 2011). The construct of exponential stability does not allow modeling these singularities, while fractional stability may be a more applicable construct with respect to describing cyclic movement patterns like gait.

The construct of fractional stability may also yield other conclusions when compared to exponential stability. As an example, λ_*S*_ was shown to increase with walking speed, indicating more unstable gait dynamics at faster walking speeds (Stergiou et al., 2004; Dingwell and Marin, 2006; England and Granata, 2007). However, these results may be biased by the inability of exponential stability to model the influence of the singularities in *d*(*t*). Faster walking speeds would likely increase the presence of high frequency changes in the trunk acceleration at heel-strike and push-off and, consequently, increase the depth of the singularities in *d*(*t*). These alterations in the singularities of *d*(*t*) with increase walking speed should theoretically lead to an increase in λ_*f*_ and β, indicating improved stability with increased walking speed. Thus, further studies might be warranted to reassess if fractional stability solves these issues with exponential stability of gait dynamics.

The presence of phase transitions within the gait cycle violates the assumptions for exponential stability. Ihlen et al. (2012a,b, 2015) repeatedly showed that stability fluctuates during the different phases of the gait cycle in both in-lab gait performance and daily-life walking. In addition, Norris et al. (2008) showed differences of the local exponential stability for different phases of the gait cycle perpendicular to the flow direction of the state space trajectory. Even though these studies provide initial evidence for phase-dependent changes in stability, they all assume that the construct of exponential stability is valid in all phases of the gait cycle. We now actually tested for the presence of exponential stability and showed that this is not the case due to singularities in *d*(*t*) created by phase transitions within the step cycle. Fractional stability was able to model these non-exponential singularities in *d*(*t*) which supports the conjecture that the gait dynamics operates in the vicinity of critical points in the function space facilitating interactions with heterogeneous and complex surroundings (Kelso, 1995).

The singularities in *d*(*t*) may emerge as artifacts of (1) high-frequency motion of the sensor relative to the person during the bouts of daily-life walking and (2) insufficient reconstruction of the state space of structurally stable systems. The singularity in *d*(*t*) are created by phase-dependent modulation above the step periodicity of 2 Hz which contain ~50% of the signal energy. It is highly unlikely that such a substantial component of the signal is entirely caused by the first abovementioned type of artifact. In the case of the second type of artifact, too small lag size *l* and dimension *m* are chosen for the state space reconstruction, which would lead to improperly unfolded gait dynamics and false recurrence of the state space trajectory which results in spurious singularities in *d*(*t*) (see Figures 4, 6 in Rosenstein et al., 1993). We used the minimum of the average mutual information function as lag size *l* and the minimum portion of false nearest neighbors as dimension *m* to ensure a properly unfolded gait dynamics and we also manipulated lag size *l* and dimension *m* further than Equations (4–6) to ensure that singularities in *d*(*t*) appeared at *t* = *l*, 2*l*, …, *ml*, irrespective of the choice of *m* and *l*. Our results indicate that the singularities in *d*(*t*) are indeed intrinsic properties of the gait dynamics created by intra-step phase-dependent modulation of trunk acceleration. Nevertheless, potential artifacts created by a combination of (1) and (2) will affect the parameters of fractional and exponential stability in an unknown way, as it will for other non-linear analyses. Thus, further studies should include in-lab 3D motion analysis of gait kinematics to isolate the signal component caused by the motion of the sensor relative to the person to investigate the influence of these artifacts and the choice of state space reconstruction methods on non-linear metrics.

The biological origin of phase-dependent changes or singularities in gait is not well known. The influence of phase transitions on gait stability has been studied as shifts in coupling strength between the knee, ankle, and hip joints angles during gait (Ihlen, 2014). These shifts showed age-related differenced during the push-off phase as being possible impairments in joint coordination in this particular phase of the gait cycle (Ihlen, 2014). The MEMD surrogate test indicates that the singularities in *d*(*t*) are generated by intra-step phasic changes in the trunk accelerations and that the fractional stability is highly dependent on the characteristics of these singularities. The MEMD surrogate test further indicates that an increase in the negative values of λ_*f*_ and decrease in operator β is generated by a loss of intra-step high frequency modulation of trunk acceleration. This suggests that the singularities in *d*(*t*) may be due to alteration in the timing of heel-strikes and push-offs. Studies with transcranial magnetic stimulation (TMS) have shown that activation of inhibitory circuits in the motor cortex leads to lower activation of plantar- and dorsiflexors during the push-off phase (Schubert et al., 1997, 1999; Capaday et al., 1999; Christensen et al., 2001; Petersen et al., 2001). Thus, increased activity of these inhibitory circuits may lead to less pronounced phase-dependent changes during foot contact and shallower singularities in *d*(*t*). However, further studies are needed to assess the relationship between TMS induced changes in parameters of fractional stability, λ_*f*_, α, and β, and corticomuscular coherence.

It remains to be shown whether this new concept of gait stability is related to balance impairment and fall risk. The present study did not compare fallers and non-fallers to validate the suggested construct of gait stability. Furthermore, the present study did not assess the relationship between the parameters of fractional stability and other fall risk factors such as medication, urinary control, vision, footwear, environmental hazards, physical and cognitive function, fall history, and fear of falling. However, defining an older person as a faller or non-faller based on retrospective or prospective self-reports is prone to recall errors, and validating a stability construct by its ability to classify or predict falls is by itself an improper validation. Further studies should use a combination of approaches, including the assessment of fractional stability in biomechanical models of human walking and associations of fractional stability with reactions to experimentally induced perturbations, to validate the parameters of fractional stability, λ_*f*_, α, and β.

The construct validity of all measures of dynamic stability in humans depends on how well they assess the ability of a walking person to recover from a perturbation. According to constructs within stability theory of dynamical systems (see Figure 1), the gait dynamics have recovered from a perturbation when the distance between the perturbed and unperturbed cycle is below a small distance ε [i.e., *d*(*t*) < ε]. As an example, it follows from the definition “*gait that does not lead to falls in spite of perturbations*” (*p*. 2, Bruijn et al., 2013) that the ε-distance in Figure 1 defines a volume around the state space trajectory of walking dynamics where no fall will occur. The construct of fractional stability indicated that all walking epochs are stable (i.e., λ_*f*_ < 0), in accordance with the definition of gait stability above. In contrast, the positive λ_*S*_ of exponential stability found for all walking epochs indicates unstable gait and would imply, by definition, that all walking epochs would pass the ε-distance and end in a fall. With the knowledge of the ε, the difference, ε–*d*(*t*) > 0, can be considered as a margin of dynamic stability and the time for *d*(*t*) to reach ε can be computed by the rate of change in *d*(*t*) quantified by λ_*f*_, α, and β. The ε separating a fall and a non-fall can be evaluated for walking models where ε can be estimated as the threshold of *d*(0), where values above this threshold diverge to a fall and values below this threshold converge to walking (Karssen and Wisse, 2009; Huang et al., 2012). The value of ε may depend on anthropometric factors, like segment mass, length and inertia, and environmental factors, like walking surface. However, ε may also be dependent on age- and disease-related changes of the neuromuscular system, which would be difficult to implement in a simple walking model. An alternative approach would be to determine ε experimentally. Series of experimentally induced perturbations of various kinds could serve to approximate ε of people walking under different contexts and with different abilities, but an extensive use of experimentally induced perturbations in older persons and patients could be considered unethical. In summary, it may be problematic to equate the mathematical construct of dynamic stability with an absence-of-fall-based definition of gait stability without knowledge of ε separating a fall from a successful balance recovery. Nevertheless, daily-life measurements where perturbations and falls occur naturally may help to tune our definition of gait stability.

The present introduction of fractional stability in gait analysis is a proof-of-concept more than an actual validation, because our application only shows the ability of the method to fit the dynamics of trunk acceleration during gait in community-dwelling older persons. Furthermore, measures of dynamic stability, including fractional stability, lack physiological and functional correlates, and require standardization in the selection of the variables and parameters for the reconstruction of the gait dynamics. Further studies should apply the present method by reconstructing the dynamics of other segments and replicate the present study on other populations including different age groups, patients with balance impairments during walking and different neurodegenerative diseases and associate fractional stability with physiological and functional characteristics of these populations.

## Conclusion

We introduced a novel approach toward gait stability, based on fractional stability, because gait dynamics with its phase transitions violates the assumptions for exponential stability. Fractional stability allows modeling the reaction distance *d*(*t*) by introducing fractional indices, α and β, of differential operator and contains the commonly used exponential stability as a special case when α = 1 and β = 1. This stability construct provided an improved model of reaction distance *d*(*t*) of gait dynamics in our sample of older community-dwelling people. Thus, fractional stability represents a more unified concept of gait stability when compared to the conventional construct of exponential stability. Our surrogate tests further indicated that the phase transitions often observed in gait dynamics are caused by intra-step variations, likely during heel strike and push off. Further validation of the fractional stability measures, λ_*f*_, α, and β, needs to assess difference in the gait dynamics of fallers and non-fallers and differences in the known stability properties of walking models. Further studies need also to assess the test-retest reliability of the fractional stability measures and investigate how these measures are affected by age, balance impairments during walking, and different neurodegenerative diseases.

## Author contributions

EI: Substantial contributed with the conception and design of the work and analysis and interpretation of data for the work and writing and revising the manuscript for important intellectual content. Kv, SB, MP, and Jv: Substantial contributed with the acquisition of data, interpretation of data, and revising the manuscript for important intellectual content. All authors have approve the version to be published and agree to be accountable for all aspects of the work in ensuring that questions related to the accuracy or integrity of any part of the work are appropriately investigated and resolved.

### Conflict of interest statement

The authors declare that the research was conducted in the absence of any commercial or financial relationships that could be construed as a potential conflict of interest.
