# Supplementary material for: Fractional Stability of Trunk Acceleration Dynamics of Daily-Life Walking: Toward a Unified Concept of Gait Stability
Source: Front Physiol. 2017 Aug 29;8:516. doi: 10.3389/fphys.2017.00516 (PMC5581839; doi:10.3389/fphys.2017.00516)
Supplement: Supplementary file 1 [file Presentation1.pdf]

Appendix A

1.0 Technical details of the surrogate generation based on the multivariate empirical mode decompositions

The MEMD surrogate generation method has the following four steps (see Fig. A1):

Step 1: Decompose the reconstructed gait dynamics, defined by Eq. 7 of the main text, into intrinsic mode functions by MEMD

Step 2: Consider the sum of high frequency components, $\mathbf{d}_1(t)$ to $\mathbf{d}_4(t)$, as the intra-step details $\mathbf{y}_{high}(t)$ and the sum of low frequency components, $\mathbf{d}_5(t)$ to $\mathbf{d}_N(t)$ together with the final residual $\mathbf{r}_N(t)$, as the inter-step periodicity $\mathbf{y}_{low}(t)$ of the gait dynamics (Fig. A1):

$$\mathbf{y}_{high}(t) = \sum_{n=1}^{4} \mathbf{d}_n(t) \qquad \mathbf{y}_{low}(t) = \sum_{n=5}^{N} \mathbf{d}_n(t) + \mathbf{r}_N(t)$$

where the original gait dynamics $\mathbf{x}(t) = \mathbf{y}_{high}(t) + \mathbf{y}_{low}(t)$.

Step 3: IAAFT surrogates were generated for the intra-step details, $\mathbf{y}_{high}(t)$.

Step 4: MEMD surrogate dynamics was obtained by adding the IAAFT surrogates and the inter-step periodicity, $\mathbf{y}_{low}(\mathrm{t})$.

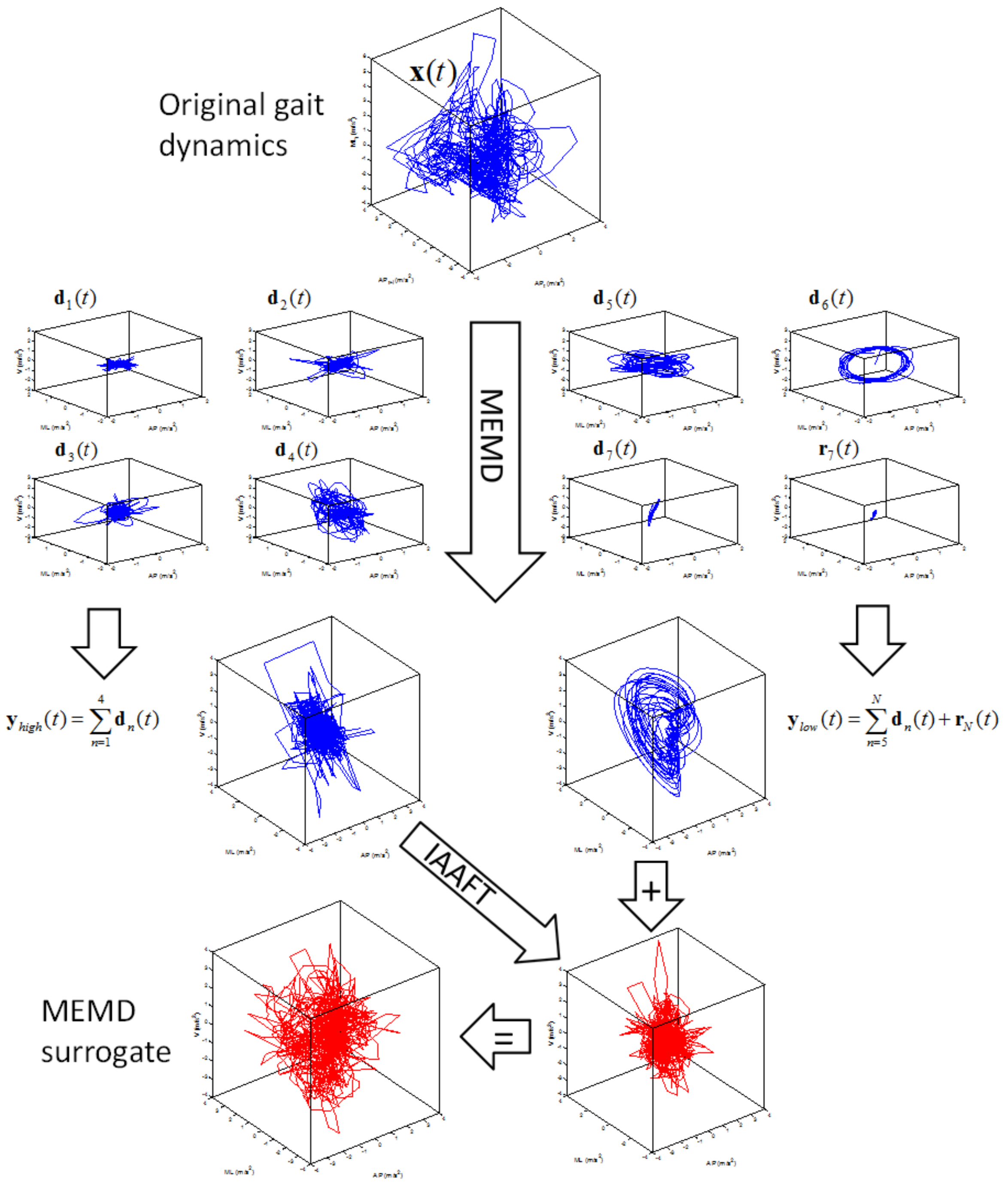

Fig. A1: Schematic representation of the generation of MEMD surrogates. The original gait dynamics in the reconstructed state space (see upper panel) are decomposed into multiple intrinsic mode functions (IMF) (see middle small panels). The high frequency intra-step details of the gait dynamics are defined as the sum of the first four IMFs (left panels) and low frequency periodicity of the gait dynamics are defined by sum of the remaining IMFs (right panels). A IAAFT surrogate (*lower right panel*) are generated for the high frequency intra-step details which preserves the distribution and power spectral density, but removes the phase-dependent

changes in these details. The MEMD surrogate is defined as the sum of the IAAFT surrogate and the low frequency periodicity of the gait dynamics (*lower left panel*).

1.1 Multivariate empirical mode decomposition (MEMD)

The following MEMD algorithm introduced by Rehman and Mandic (2010) was used in the present study (see acknowledgment):

Step 1: Generate a Hammersley sequence-based point set on a $3m - 1$ dimensional sphere where m is the number of lags in the state space reconstruction method (m = 1, 2, and 3 in the present study) .

Step 2: Compute the projection $p_{\theta_k}(t)$ of the gait dynamics x(t) = (or residual $\mathbf{r}(t)$ or $\mathbf{d}(t)$ for iterative steps) along the unit direction vectors θ_k of the $3m - 1$ dimensional sphere.

Step 3: Find the time instant t_{θ_k} that corresponding to the maxima $p_{\theta_k}^{\max}(t)$ of $p_{\theta_k}(t)$ along all $k = 1, 2,\ldots,3m - 1$ dimensions.

Step 4: Obtain the envelope curves, $e_{\theta_k}(t)$, by component-wise spline interpolations between all time instants t_{θ_k} of $p_{\theta_k}^{\max}(t)$.

Step 5: Compute the mean $\mathbf{m}(t)$ of all envelope curves, $e_{\theta_k}(t)$, across all $3m - 1$ directions of the sphere by the following equation:

$$\mathbf{m}(t) = \frac{1}{3m-1}\sum_{k=1}^{3m-1} e_{\theta_k}(t) \qquad \text{(A6)}$$

Step 6: The first series of details $\mathbf{d}_1(t)$ around the mean $\mathbf{m}_1(t)$ is defined as $\mathbf{d}_1(t) = \mathbf{x}(t) - \mathbf{m}_1(t)$. If $\mathbf{d}_1(t)$ satisfies the selected stopping criteria, then $\mathbf{d}_1(t)$ is defined as an intrinsic mode

function (IMF) and Step 2 to 5 is performed on first residual, $\mathbf{r}_1(t) = \mathbf{x}(t) - \mathbf{d}_1(t)$. The second IMF is defined as $\mathbf{d}_2(t) = \mathbf{r}_1(t) - \mathbf{m}_2(t)$ with residual $\mathbf{r}_2(t) = \mathbf{r}_1(t) - \mathbf{d}_2(t)$. Consequently, the *n*th IMF is defined as $\mathbf{d}_n(t) = \mathbf{r}_{n-1}(t) - \mathbf{m}_n(t)$ with residual $\mathbf{r}_n(t) = \mathbf{r}_{n-1}(t) - \mathbf{d}_n(t)$. This iterative shifting procedure (i.e., Step 2 to 5) is continued until two maxima $p_{\theta_k}^{\max}(t)$ of the projection $p_{\theta_k}(t)$ in Step 3 can no longer be found. If $\mathbf{d}_n(t)$ do not satisfy the stopping criteria, then step 2 to 5 is performed as a iterative procedure on $\mathbf{d}_n(t)$ until the stopping criteria is met and a IMF is defined. The stopping criteria used in the present study is similar to the stopping criteria proposed by Rilling et al. (2003) except the criteria of equality between the number of zero crossings and number of maxima. The sum of all IMFs and the final residual, $\mathbf{x}(t) = \sum_{n=1}^{N} \mathbf{d}_n(t) + \mathbf{r}_N(t)$, becomes the gait dynamics $\mathbf{x}(t)$.

1.2 Iterated amplitude adjusted fourier transform (IAAFT)

IAAFT surrogate was generated for the intra-step details of the gait dynamics, $\mathbf{y}_{high}(t)$, defined by Eq. A1 by following five steps (Schreiber and Schmitz, 1996):

Step 1: Store the rank ordering of the amplitudes of $\mathbf{y}_{high}(t)$.

Step 2: Fast fourier transform $\mathbf{y}_{high}(t)$ and store the obtained spectral amplitudes.

Step 3: Randomly shuffle $\mathbf{y}_{high}(t)$.

Step 4: Initiate an iterative procedure where:

1) The spectral amplitudes of shuffled time series are obtained by a fast Fourier transformation.

2) The obtained spectral amplitudes of the shuffled time series are substituted with the stored spectral amplitudes of $\mathbf{y}_{high}(t)$.

3) The surrogate series are obtained by an inverse fast Fourier transformation.

4) The amplitudes of obtained surrogate series are ranked as in $\mathbf{y}_{high}(t)$.

In the present study, 1 to 4 is iterated a maximum of 500 times to obtain a saturation effect as described by Schreiber and Schmitz (1996) where the surrogate series had equal power spectral density and probability density function as $\mathbf{y}_{high}(t)$, but where the phase-dependent changes in $\mathbf{y}_{high}(t)$ are removed.

Appendix B

Matlab code for numerical estimation of fractional stability

The following Matlab function, `frac_stab`, estimate α, β, λ_f and C of fractional stability defined by Eq. 5. The Matlab code is based on, `div_frac_calc`, used to calculate reaction size d(t) in Rispens et al. (2015) and `mlf` developed by Podlubney which computes the generalized Mittag-Leffler function in Eq. 6. `mlf` and is available at https://www.mathworks.com/matlabcentral/fileexchange/8738-mittag-leffler-function/content/mlf.m. `mlffit2` developed by Podlubney et al. (2012) could be used instead of `gml_stability_fit.` `mlffit2` is available at http://www.mathworks.com/matlabcentral/fileexchange/32170-fitting-data-using-the-mittag-leffler-function/content/mlffit2.m

```
function [alfa,beta,lambda_f,C,dt_upsamp,dt_fit,t]=
frac_stab(Acc_all,mean_step_time,lag_size,lag_numb,singular_numb,fs,Fig)

%Estimation of fractional stability defined by Eq. 5 in Ihlen et al.
%(submitted)

%INPUT PARAMETERS-----------------------------------------------------------
%Acc_all_______________[n,3] matrix where Acc_all(:,1) are acceleration
%                       in AP direction, Acc_all(:,2) the same in ML
%                       direction and Acc_all(:,3) the same in V direction.
%mean_step_time_________Mean step time for Acc_all or the mean periodicity
%                       of the signal.
%lag_size_______________Chosen lag size in number of samples for the state
%                       space reconstruction
%lag_numb_______________Number of lag used in the delayed coordinate
%                       embedding
%singular_numb__________Number of singularities in d(t)to be used in the
%                       estimation of fractional stability in Eq. 5
%fs_____________________Sampling frequency
%Fig____________________0/1-flag where 1 displays the fit of the fractional
%                       stability (Eq. 5)
%
%OUTPUT PARAMETERS----------------------------------------------------------
%
%alfa___________________Vector with m indices where m = singular_numb
%beta___________________Vector with m indices where m = singular_numb
%lambda_f_______________Vector with m indices where m = singular_numb
%C______________________Vector with m indices where m = singular_numb
%dt_upsamp______________Upsampled version of d(t) between singularities.
```

```
%                        [n,m] matrix where n is sample number and m is the
%                        number of singularities.
%dt_fit________________The fitted fractional stability (Eq. 5) to d(t).
%                        Same [n,m]matrix as a above
%t_____________________Time indexes for dt_upsamp and dt_fit. Same [n,m]
%                        matrix as above.
%
%--------------------------------------------------------------------------

%Reconstruction of state space defined by lag-numb and lag_size.
%The dynamics are reconstructed for the AP, ML, and V direction combined---
if singular_numb>lag_numb,
   disp('singular_numb should be equal or lower than lag_numb')
end
X_ap=Acc_all(:,1);
X_ml=Acc_all(:,2);
X_v=Acc_all(:,3);

N_ss = size(X_ap,1)-(lag_numb-1)*lag_size;
state_ap=nan(N_ss,lag_numb*size(X_ap,2));
state_ml=nan(N_ss,lag_numb*size(X_ml,2));
state_v=nan(N_ss,lag_numb*size(X_v,2));

for dim=1:size(X_ap,2),
    for delay=1:lag_numb,
        state_ap(:,(dim-1)*lag_numb+delay)=X_ap((1:N_ss)'+(delay-
        1)*lag_size,dim);
        state_ml(:,(dim-1)*lag_numb+delay)=X_ml((1:N_ss)'+(delay-
        1)*lag_size,dim);
        state_v(:,(dim-1)*lag_numb+delay)=X_v((1:N_ss)'+(delay-
        1)*lag_size,dim);
    end
end

state_space=[state_ap,state_ml,state_v];

%Calculation of reaction distance d(t) by Rosenstein et al. (1993) method--
[dt]=div_frac_calc(state_space,mean_step_time,fs);

%Identification of singularities in d(t)------------------------------------
[pks,locs0]=findpeaks(-dt,'MinPeakDistance',lag_size-2);
locs=[1,locs0];

%Resampling and estimation of fractional stability (Eq. 5) between each
singularity
for n=1:singular_numb,
    dt_upsamp(:,n)= interp1(1:length(locs(n):locs(n+1)),
dt(locs(n):locs(n+1)),linspace(1,length(locs(n):locs(n+1)),50),'pchip');
    t1=linspace(1,length(locs(n):locs(n+1)),50)./fs;
    t(:,n)=linspace(locs(n),locs(n+1),50)./fs;
    par=[2; 2; dt_upsamp(1,n); -1];
    [c0,dt_fit0]=gml_stability_fit(dt_upsamp(:,n)',t1,par);
    dt_fit(:,n)=dt_fit0;
    alfa(n)=c0(1);
    beta(n)=c0(2);
    lambda_f(n)=c0(4);
    C(n)=c0(3);
end
```

```
%Figure output-----------------------------------------------------------------------
if Fig==1,
   figure;
   for n=1:singular_numb,
       plot(t(:,n),dt_upsamp(:,n),'bo-');hold all
       plot(t(:,n),dt_fit(:,n),'r-','LineWidth',2);
   end
end
%-----------------------------------------------------------------------------
function [d_t]=div_frac_calc(state_space,mean_step_time,fs)

%computation of reaction size d(t) according to method proposed by
%Rosenstein et al. (1993). The script is a modified version of a script
%div_calc developed by Sietse Rispens used in Rispens et al. (2015).

period_sec = mean_step_time/fs;
WindowLen = floor(mean_step_time);
ws_sec=WindowLen/fs;

[m,n]=size(state_space);
ws=round(ws_sec*fs);
period=round(period_sec*fs);
mcompletewindow = m-ws+1;
statecw = state_space(1:mcompletewindow,:);
divergence_sum=zeros(1,ws);
divergence_count=zeros(1,ws);
diff_state = statecw*0;
diff_state_sqr = diff_state;
diff_total = zeros(size(diff_state,1),1);
for i = 1:mcompletewindow
    if ~isnan(state_space(i,:))
        start=round(max([1,i-period+1]));
        stop=round(min([mcompletewindow,i+period-1]));
        for j=1:n,
            diff_state(:,j) = statecw(:,j)-statecw(i,j);
            diff_state_sqr(:,j)=diff_state(:,j).^2;
        end
        diff_total(:,1)=sum(diff_state_sqr,2);
        diff_total(start:stop,1)=NaN;
        [mini,index]=min(diff_total);
        if i+ws>m || index+ws>m
            % do not use these data
        else
            divergence_sum = divergence_sum + sqrt(sum((state_space(i:i+ws-
            1,:)-state_space(index:index+ws-1,:)).^2,2))';
            divergence_count = divergence_count + 1;
        end
    end
end

d_t = (divergence_sum./divergence_count);
```

```
function [par_fit,d_fit]=gml_stability_fit(d,t,par0)

fun = @(par,t)gml_stability(par,t);
lb=[0,0,0,-100000];
ub=[5,3,1000,100000];
options = optimoptions(@lsqcurvefit,'Algorithm','trust-region-
reflective','TolFun',1.0000e-
14,'MaxFunEvals',3000,'MaxIter',3000,'Display','none');
par_fit = lsqcurvefit(fun,par0,t,d,lb,ub,options);
d_fit=gml_stability(par_fit,t);



function d=gml_stability(para,t)

d=para(3)* t.^(para(2)-1) .* mlf(para(1), para(2), para(4)*t.^para(1),6);
```
